# Theory and applications of differential scanning fluorimetry in early-stage drug discovery

**DOI:** 10.1007/s12551-020-00619-2

**Published:** 2020-01-31

**Authors:** Kai Gao, Rick Oerlemans, Matthew R. Groves

**Affiliations:** Structure Biology in Drug Design, Drug Design Group XB20, Departments of Pharmacy, University of Groningen, Groningen, The Netherlands

**Keywords:** Thermal stability, Folding, Unfolding, Refolding, Fluorimetry, Ligands screening, Crystallization, Buffer optimization

## Abstract

Differential scanning fluorimetry (DSF) is an accessible, rapid, and economical biophysical technique that has seen many applications over the years, ranging from protein folding state detection to the identification of ligands that bind to the target protein. In this review, we discuss the theory, applications, and limitations of DSF, including the latest applications of DSF by ourselves and other researchers. We show that DSF is a powerful high-throughput tool in early drug discovery efforts. We place DSF in the context of other biophysical methods frequently used in drug discovery and highlight their benefits and downsides. We illustrate the uses of DSF in protein buffer optimization for stability, refolding, and crystallization purposes and provide several examples of each. We also show the use of DSF in a more downstream application, where it is used as an in vivo validation tool of ligand-target interaction in cell assays. Although DSF is a potent tool in buffer optimization and large chemical library screens when it comes to ligand-binding validation and optimization, orthogonal techniques are recommended as DSF is prone to false positives and negatives.

## Introduction

Biophysics drives modern drug discovery efforts, allowing rapid and high-throughput data acquisition to screen through large compound libraries in an effort to identify new bioactive molecules. An important component of this biophysics armory is the thermal shift assay, also commonly known as differential scanning fluorimetry (DSF) (Semisotnov et al. 1991). DSF is a cost-effective, parallelizable, practical, and accessible biophysical technique widely used as a method to track both protein folding state and thermal stability. It provides a reliable tool to examine protein unfolding by slowly heating it up in a controlled environment. By measuring the corresponding changes in fluorescence emission upon temperature increase, the process of protein denaturation can be monitored. Since changes in sample behavior through complex formation with even weakly binding ligands affect protein thermal stability, the technique has seen many successful applications and has been used in different ways over recent years. It has been utilized primarily as a drug discovery method to identify promising lead compounds for a number of target proteins for decades (Pantoliano et al. 2001). Another major application for DSF is in protein buffer optimization, identifying optimal conditions for storage, assay screening, and crystallization. By screening sparse matrix conditions, encompassing different buffer systems that cover a wide range of pH, additives, and salt concentrations, optimal buffer components can be identified for each individual protein. This has been shown to increase the success rates of protein crystallization in past decades (Huynh and Partch 2015). More recently, DSF has also been applied to the challenge of sample preparation, with two publications demonstrating that suitable screening approaches can be used to identify and optimize sample refolding buffers—allowing significantly cheaper access to the amounts of protein sample required to support high-throughput screening campaigns (Biter et al. 2016; Wang et al. 2017). Finally, a very recent development has shown that DSF is able to provide reliable data in complex solutions, such as unpurified chemical reactions. This is an exciting development, as the production and purification of chemical entities are a major bottleneck in any screening campaign.

While the robustness of the DSF method and its broad applicability in both sample preparation and screening has led it to become an important biophysical tool in drug discovery, it is important to bear its limitations in mind. This is particularly true when designing a screening campaign, as such a campaign should contain orthogonal screening options that are not susceptible to similar limitations—in order to minimize both false positives and false negatives.

In this review, we will provide a theoretical background of DSF as well as examples of its use in the various aspects of drug discovery introduced above—including the latest applications of DSF by ourselves and other researchers. We will also attempt to place DSF within the variety of biophysical methods currently used in screening campaigns and highlight areas of overlap or mutual limitations.

## Theory of differential scanning fluorimetry

In 1997, Pantoliano et al. (1997) introduced a new thermal shift assay system used in the screening of combinatorial libraries against different receptor proteins. Compared with conventional methods of the time, such as those based on calorimetry and spectral technologies (Bouvier and Wiley 1994; Weber et al. 1994), the newly developed system could implement high-throughput screening instead of assaying a single condition at a time. The custom-designed 96- or 384-well plates and fluorescence readout apparatus could easily monitor protein unfolding in multiple conditions, with different ligands and/or at different ligand concentrations in a single experiment. This helped researchers overcome a lot of cumbersome, slow, and labor-intensive work required by traditional methods. Rather than the need for a dedicated device, many labs already possess (or have access to) real-time polymerase chain reaction (RT-PCR) equipment that allows for fluorescence measurements over a controlled temperature range. Access to such equipment, the development of more sensitive dyes, and improved protocol design drove the use of DSF (Niesen et al. 2007).

**Fig. 1:**
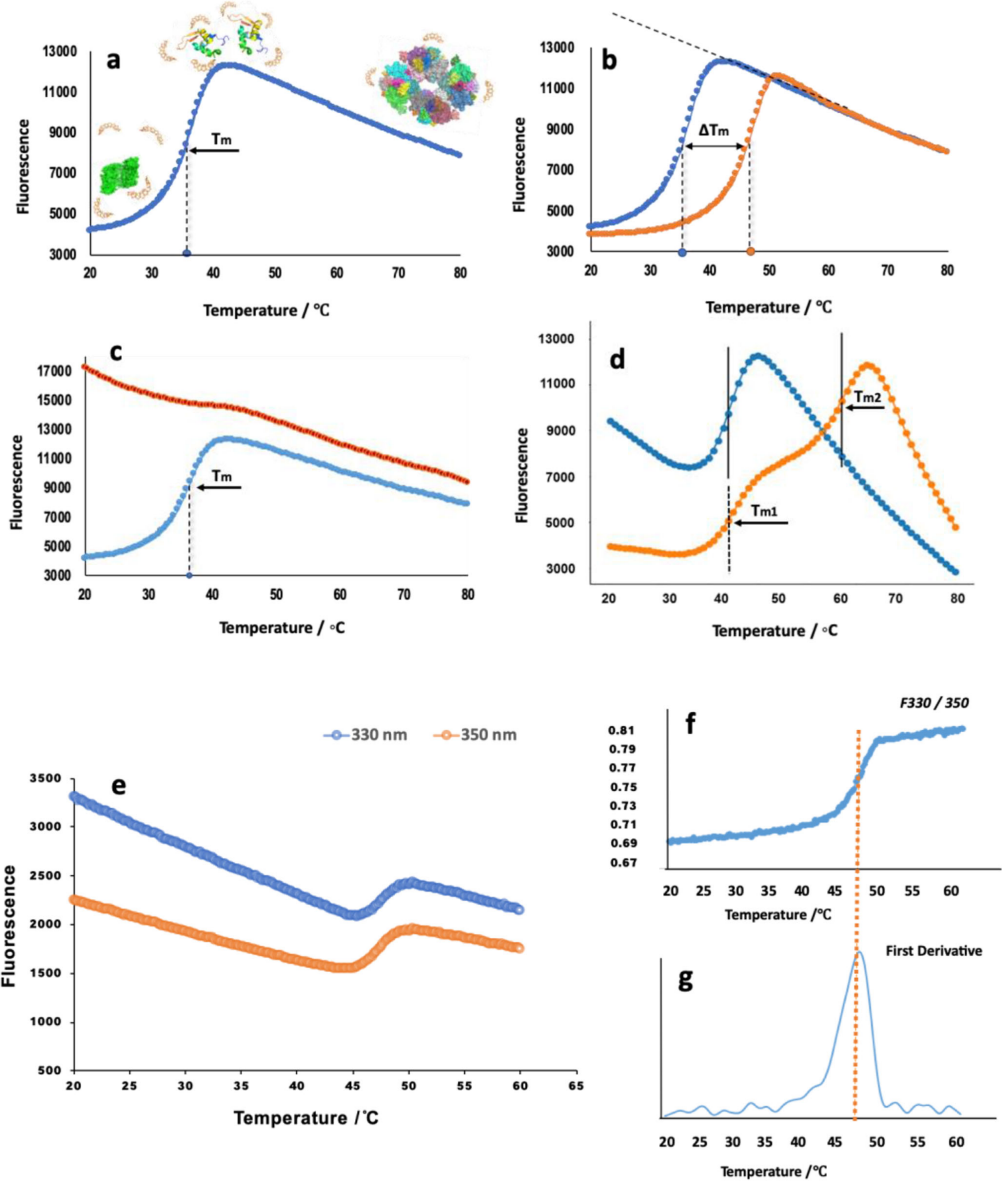
**a** Typical thermal denaturation profile of a protein sample. Fluorescence emission changes with the temperature. The sigmoidal curve indicates the cooperative unfolding status of the protein from trace amounts of SYPRO Orange (yellow) bound to the native protein (green). The peak indicates that all proteins are unfolded to linear peptides or that the hydrophobic core is exposed to SYPRO Orange. Multiple mechanisms exist for the reduction in fluorescence after the peak, including temperature-driven decrease in the binding constant of the dye (so less dye is bound to the protein), the pocket binding the dye being more mobile (allowing for more quenching by solvent); the dye itself is more mobile such that the degree of planarity required for electron conjugation/aromatic character is lessened and protein aggregation and dye dissociation through the exclusion of the dye from hydrophobic cores. The midpoint of the transition curve is the melting temperature (*T*_m_). **b** DSF curve showing the unfolding status of a target protein in the absence (blue) and presence (orange) of a ligand. The difference in the melting temperature indicated as Δ*T*_m_. **c** Sample with high background fluorescence at the beginning at lower temperature (red) compared with a typical well-folded sample (blue) in the DSF assay. Improperly folded, aggregated, denatured protein or hydrophobic area such as a lipid bilayer exposed to the dye will cause high background at low temperatures. **d** Multiple transitions appearing during the heating process can be caused by different domains, aggregation increasing with temperature, or ligands that stabilize a portion of the protein sample (orange); typically one *T*_m_ similar to the native protein is accompanied by one or more *T*_m_ at a higher temperature during the denaturation. **e**–**g** Overview of NanoDSF. **e** Intrinsic fluorescence of tryptophan is measured at both 330- and 350-nm wavelengths and plotted versus temperature from 20 to 60 °C during unfolding. **f** F330/350 fluorescence ratio intensity of tryptophan plotted against temperature. **g** The melting temperature is calculated by the first derivative of the F330/350 plots, with the sample given here showing a *T*_m_ of 48 °C. All the figures above represent thermal unfolding curves of the menin protein and are obtained from DSF experiments conducted in our lab. The experiments were performed by using either the Bio-Rad CFX96 Real-Time PCR system or the NanoTemper Prometheus NT.48 system. Curves were plotted from the fluorescence data using Excel

Proteins are in a thermodynamic equilibrium between folded and unfolded states (Bowling et al. 2016). An increase in energy of the environment (i.e., increase in temperature) pushes a protein toward the unfolded state which, when quantified, allows for the determination of the melting temperature (*T*_m_), defined as the temperature at which 50% of a protein sample is in folded and 50% is in an unfolded state (Lo et al. 2004) (Fig. 1a). A change in the protein environment (including pH, ionic strength, or the presence of specific anions or cations) and/or complex formation with other molecules can stabilize a protein through a reduction of the Gibbs free energy of the complex, resulting from the creation of new molecular interactions (hydrogen bonds, van der Waals interactions, etc.) or conformational reordering of the target protein. This increase in the Gibbs free energy results in an increase in thermal stability and thereby an increase in the melting temperature (*T*_m_). Measurements of the *T*_m_ of a protein in the presence and absence of environment changes or ligands result in an estimate of the thermal shift (Δ*T*_m_) deriving from these differences (Scott et al. 2016) (Fig. 1b). This shift is typically an indicator of complex formation and/or thermal stabilization. However, it should be borne in mind that while the resulting temperature shift is directly related to the change in the Gibbs free energy, it is a measurement deriving from both binding interactions and any resulting conformational changes in the target protein, and as the thermal stability profile is generated over a temperature range, it is difficult to generate a reliable room temperature dissociation constant (*k*_d_ = exp −∆G/*kT*; *k* = Boltzmann’s constant and *T* = thermodynamic temperature) directly from Δ*T*_m_. However, solely concentrating on *T*_m_ may mean that other systemic and thermodynamic information about protein stability can be lost. The propensity of the protein to aggregate in certain conditions is one such factor. An environmental change could result in a difference in aggregation behavior but leaves the *T*_m_ unchanged. For an in-depth review on this topic, please see Wakayama et al. (2019).

In order to monitor the thermal unfolding transition of target protein in a suitably sensitive but precise way, fluorescence has been used as the response signal. There are two main sources of this fluorescence in use today that may be broadly classed as (i) extrinsic fluorescence and (ii) intrinsic fluorescence.

## Extrinsic fluorescence

The fluorescence of extrinsic fluorescent dyes is sensitive to their environment. Typically such dyes are quenched in aqueous solutions with proteins in their native folded state and provide a fluorescence signal only when the target protein begins to unfold. This unfolding allows the freely diffusing dye to interact with the exposed residues of the hydrophobic core (Fig. 1a). This approach relies on the following assumptions (in rough order of frequency as experienced by the authors):

- The target proteins do not possess significant hydrophobic patches on their exposed surfaces, the presence of which would lead to increased background in fluorescence (Fig. 1c).
- The protein is in a stable state at the beginning of the experiment, and DSF experiments using extrinsic dyes are typically performed at concentrations of 0.1–0.5 mg/ml (0.01–0.1 μM). Aggregation and/or sample instability may lead to the presence of multiple species of target protein within the experiment, both leading to increased fluorescence background from any conformational variability and resulting in variable thermal stability profiles of the different order oligomers (Fig. 1c).
- The target protein shows no significant binding interaction(s) with the dye in use—resulting in the shielding of the dye from the aqueous environment prior to protein unfolding and a resulting increase in fluorescence background.
- The target protein is composed of a single domain, as the unfolding of distinct domains is likely to occur with different *T*_m_ values resulting in a complex thermal stability profile (Fig. 1d). However, while the profile might be more complex, it is often easier to differentiate between the signals from multiple domains and this can provide valuable information as seeing a *T*_m_ shift more strongly in a specific domain can provide information about a potential binding site.
- No major structural rearrangements of the target protein are provoked by an increased temperature prior to its unfolding, although in such cases, deconvolution of the thermal stability profile may still be possible.
- The sample and dye do not chemically react with other components present in the experiment over the temperature range used.

## Dyes in common usage

There are many commercial dyes available (Hawe et al. 2008). Dyes such as bis-ANS and Nile Red have been used for decades; the extrinsic dyes are summarized in Table 1. However, these dyes all possess a significant background in the presence of folded proteins. To date, the most favored dye for DSF is SYPRO Orange, mainly owing to its high signal-to-noise ratio (Niesen et al. 2007), as well as its relatively long excitation wavelength (near 500 nm). This minimizes the interference of most small molecules as these typically have absorption maxima at shorter wavelengths.

**Table 1:** Overview of extrinsic fluorescence dyes applied in protein characterization

Dye	Molecular formula	Application	Excitation (nm)	Emission (nm)	Reference
bis-ANS	C32H22K2N2O6S2	Hydrophobicity unfolding/folding aggregation	395	470–530	Grillo et al. (2001)
Nile Red	C20H18N2O2	Hydrophobicity unfolding/folding aggregation	450	590–665	Greenspan et al. (1985)
SYPRO Orange	C28H42N2O3S	Hydrophobicity unfolding/folding aggregation	488	500–610	Lo et al. (2004)
DCVJ	C16H15N3	Viscosity of protein environment rigidity	433	480–530	Menzen and Friess (2013)
CCVJ	C16H16N2O2	Viscosity of protein environment rigidity	435	480–505	Rumble et al. (2012)
ThT	C17H19ClN2S	Fibrillation Aggregation	450	460–600	Nielsen et al. (2001)
ProteoStat	C45H62I2N4^a^	Protein aggregation	488	600	McClure et al. (2018)
CPM	C16H14N2O4	Hydrophobicity Cysteine related	387	463	Alexandrov et al. (2008)

^a^Abstracted from patent (Patton et al. 2013)

## Intrinsic fluorescence

Another source of fluorescence is from the protein sample itself. In 2010, Schaeffer’s team reported a new method, using green fluorescent protein (GFP) to quantify the stability of a target protein (Moreau et al. 2010). In these experiments, a GFP tag was fused to a protein of interest through a peptide linker and used as a reporter system for protein unfolding and aggregation. The fluorescence signal from the GFP changes based on its proximal environment, meaning its signal can be used to monitor the unfolding of the protein it was linked to. Since GFP only starts losing fluorescence around 75 °C, this approach suits a large number of proteins which are significantly less stable than GFP (Moreau et al. 2010). While this is potentially an elegant solution to remove reliance on a fluorescent dye reporter, there do remain a number of limitations:

- The potential for interaction between GFP and the target of interest influencing the target protein conformation, thereby introducing a bias into the measured interactions with ligands.
- The potential for a GFP-linked domain to influence the oligomeric state of the target protein—either promoting or inhibiting assembly—with a similar effect on the target protein conformation.
- This approach is unsuitable for protein targets that have a similar *T*_m_ to that of GFP—in which case the unfolding signal of the target protein will be masked by that of GFP
- Ligands that may result in a significant elevation of the target-to-ligand complex *T*_m_ will not be clearly observed due to a similar masking effect.
- This approach is unable to directly distinguish between compounds that interact with GFP and those that interact with the target protein, although this can be addressed through the use of a GFP only control.

In 2014, a label-free DSF technique marketed as nanoDSF was developed (Alexander et al. 2014). This approach removes the requirement for an extrinsic dye or fusion tag, instead of relying on the change of intrinsic tryptophan fluorescence at 330 nm and 350 nm (Fig. 1e). Unfolding/denaturation results in a change in the microenvironment polarity around tryptophan residues, leading to a redshift of fluorescence (Ghisaidoobe and Chung 2014). In this approach, the *T*_m_ can be determined by measuring the ratio of the fluorescence at 330 nm and 350 nm against temperature (Fig. 1f, g). The commercial instrument Prometheus NT.48 (NanoTemper Technologies, Munich) allows a rapid analysis for both ligand screening and buffer composition optimization and, unlike the previous approaches, allows for measurements to be made in detergent-containing solutions—a prerequisite for DSF application to membrane proteins. Due to the nature of extrinsic dyes, which can bind (and fluoresce) in the presence of lipid bilayers and detergent micelles, conventional DSF cannot handle the detergent selection for membrane protein solubilization. The dye-free nanoDSF avoids this problem by using intrinsic fluorescence. Another benefit to intrinsic fluorescence is the ability to observe the transition both from folded to unfolded states and from unfolded to folded states. This allows for the detection of hysteresis (Andrews et al. 2013). The presence of hysteresis can provide information about protein stability (Mizuno et al. 2010). Due to the presence of dye, this is not possible when using an extrinsic fluorescence approach. However, the intrinsic fluorescence method also has several key limitations:

- The number of tryptophan residues in the target protein amino acid sequence needs to be considered before adopting this approach, since at least one tryptophan has to be present and the ratio of tryptophan present in the target protein sequence is the limiting factor to detect an unfolding signal.
- Experiments that result in complex populations in the thermal profile (e.g., presence of both bound and unbound states—see below) may not be successfully identified due to signal sensitivity.
- This approach requires a significantly larger investment for the associated equipment.

Finally, it should be clearly borne in mind that all DSF approaches are sensitive to the intrinsic fluorescence properties of the molecules present in the screen under examination, which can result in a wide variation in the background of thermal profiles—resulting in false negatives. While the use of extrinsic dyes alleviates this to some extent, as the role of the dyes in use is to significantly amplify the unfolding signal, there still remains the potential for screening components to interact with the reporting dye.

## Recent applications of DSF

### Ligand screening in drug discovery

Determining the interaction between receptors and members of a small molecule library is addressed by detecting and measuring changes in the physicochemical properties of any ligand-to-target complexes that are formed. Quantitative information arising from receptor-ligand complex formation can then drive the development process through structure-activity relationships (SAR). In the last few years, great efforts have been expended to find a general and universally applicable approach to detect binding (and ideally estimate binding affinity, *K*_d_) between biomolecule receptors and small molecule ligands. As a result, many new biophysical technologies have emerge, briefly:

- Differential scanning calorimetry (DSC), which monitors the change in heat capacity of protein samples undergoing temperature-induced melting transitions in the presence and absence of small molecule ligands (Pantoliano et al. 1989).
- Isothermal titration calorimetry (ITC), which compares the temperature differences between a reference and receptor solution to quantify the kinetic parameters of binding (Herrera and Winnik 2016).
- Surface plasmon resonance (SPR), which records the angular shift of polarized light reflected from a metal film, containing a surface-immobilized target leading to changes in refractive indices upon association and dissociation of ligand (Navratilova and Hopkins 2010).
- Microscale thermophoresis (MST), which detects the thermophoretic behaviors of receptors in the presence of ligands under heating in capillaries (Wienken et al. 2010).
- NMR-based chemical shift screening, ligand-based or protein-based NMR monitors chemical shift perturbation induced by ligands; thereby, both *K*_d_ and the structural conformation of complexes can be determined.
- X-ray crystallography–driven fragment optimization based on the electron density of ligands, providing interaction details at atomic resolution.
- Mass spectrometry–based approaches, protein samples, and bound ligands are ionized preserving non-covalent interactions. Subsequently, the mass of protein and ligands can be acquired with high accuracy (multiple instances are provided in the table below).
- Biolayer interferometry (Wartchow et al. 2011) provides similar binding information to that obtained by SPR, with advantages in signal stability arising from the use of interferometry patterns.

**Table:** 

Method	Principle	Advantages	Limitations	Ref
Ligand-observed NMR	Shift change in magnetic state of ligand due to binding	Many fragments can be tested simultaneously	Uses a lot of protein. Limited to fragments with fast exchange with target	Krimm (2017)
Protein-observed NMR	Protein NMR peak shift induced by binding	Able to determine binding site. Titration possible to determine *K*_D_	Requires large amounts of protein. Limited throughput	Krimm (2017)
X-ray crystallography	X-ray diffraction of cocrystallized protein-ligand complex or soaked apo-crystal	Provides structural information of ligand-binding mode and interactions with the target. Enables use of computational methods of hit optimization	Needs good-quality crystals. Not all the ligands can acquire cocrystal structures with protein target. Needs synchrotrons to obtain x-ray diffraction data. Requires large amounts of ligand	Badger (2012); Patel et al. (2014)
SPR	Refractive index change due to ligand binding to immobilized target on sensor	Able to easily obtain *K*_D_ and other kinetic data. Uses very little protein	Protein needs to be able to be immobilized	Neumann et al. (2007); Chavanieu and Pugnière (2016); Huber et al. (2017)
DSF	Thermal stability of protein is increased due to fragment binding	High throughput, cheap materials, equipment easy to use and widely available	Many false positives and negatives. Typically only provides a yes/no answer. Requires a dye or intrinsic fluorescence	Lo et al. (2004); Douse et al. (2015); Bai et al. (2019)
Isothermal titration calorimetry (ITC)	Heat of the system changes upon binding event	Thermodynamic and binding properties of protein—fragment interaction can directly be obtained. Label-free	Uses large amount of protein; low throughput	Chaires (2008); Ladbury et al. (2010); Renaud et al. (2016)
Differential scanning calorimetry (DSC)	Amount of heat required to increase temperature of sample changes upon binding	Highly sensitive method. Label-free	Uses a lot of protein. Low throughput	Cooper (2003); Bruylants et al. (2005); Erlanson et al. (2016)
Native mass spectroscopy (MS)	Mass detection of protein-ligand complex in gas phase	Highly sensitive method. Uses very little protein. Label-free. Provides large amount of information, binding affinity, stoichiometry	Protein has to be stable in ESI buffer	Qin et al. (2015); Pedro and Quinn (2016); Ren et al. (2019)
Size exclusion chromatography (SEC) MS	Incubation of protein in fragment mixture then separation of bound from unbound molecules by SEC, followed by MS detection	Very high throughput. Easy to perform technique requiring simple LC-MS	Potential for false negatives for low affinity binders; these can easily get lost during the SEC step	Qin et al. (2015); Chan et al. (2017); Ren et al. (2019)
Weak affinity chromatography (WAC) MS	Separation of molecules by affinity to immobilized receptor on the WAC column followed by MS detection	Easy method to use. High throughput possible by using fragment mixtures	Protein needs to be immobilized on the column	(Duong-Thi et al. 2011; Chan et al. 2017; Ohlson and Duong-Thi 2018)
Hydrogen-deuterium exchange (HDX) MS	Ligand binding affects deuteration rate of protein residues. Which is detectable by mass	Binding site can directly be elucidated and gives information about protein conformational changes	Low throughput and expensive	Chan et al. (2017); Marciano et al. (2014)
Microscale thermophoresis (MST)	Change in the molecular motion of the target in a temperature gradient due to ligand binding	Measurements can be performed in native buffers. Allows for *K*_D_ determination	Target needs to be labeled or have sufficient intrinsic fluorescence. Relatively low throughput	Linke et al. (2016); Rainard et al. (2018)
Affinity capillary electrophoresis (ACE)	Change in electrophoretic mobility of the ligand due to binding to target (in solution)	High throughput. Sensitive method. Uses small amounts of protein and ligand. Both target and ligand are free in solution	Requires detectable probe molecule or detectable fragments	Xu et al. (2016); Austin et al. (2012); Farcaş et al. (2017)
Biolayer interferometry (BLI)	Interference pattern change due to ligand binding to immobilized target on biolayer	Can obtain *K*_D_ and other kinetic parameters. Uses a small amount of protein	Immobilization of protein is required	Wartchow et al. (2011)

With the advent of modern advances in bioinformatics and proteomics, many new disease targets have been identified (Lippolis and Angelis 2016). In parallel chemical synthesis, methods are more advanced and refined, being capable of rapidly producing large libraries of diverse compounds. A particularly important subgroup of these methods is those that are compatible with multicomponent reaction (MCR) chemistry (e.g., the UGI reaction) which can generate large libraries of highly specific compounds in a short amount of time. However, the pace at which chemical libraries could be screened using conventional techniques such as NMR and ITC often could not keep up with the speed that the libraries were being created, or the numbers of discrete molecules contained in these libraries.

Modern DSF is well placed to address these large and diverse libraries, as it utilizes a real-time PCR machine to rapidly screen multiple molecules at once against the target protein, meaning it can handle the high throughput of compounds much better than many other technologies. With relatively low consumption of protein sample, 96, 384, or 1536 ligands can be analyzed in a single screen that takes ~ 1 h and provides qualitative binding information; it is well-suited for high-throughput library screening. This efficient workflow makes it possible to judge and rank potential binding affinity.

In 2001, Pantoliano introduced a DSF-based high-throughput methodology for a variety of therapeutic target proteins (human α-estrogen receptor (ESR), bacteriorhodopsin, human α-thrombin, bovine liver dihydrofolate (DHFR), the extracellular domains of the fibroblast growth factor receptor-1 (D(II)-D(III)FGFR), and the enzyme PilD; Pantoliano et al. 2001). These targets were screened against various small molecules from combinatorial libraries, including known binding ligands. Experiments showed that the *K*_d_ calculated from Eq. (1) based on the *T*_m_ values obtained experimentally gives very similar values to those previously acquired by other techniques. For example, tamoxifen inhibits the ESR antagonist with an IC_50_ value reported as 0.42 μM (Bolger et al. 1998), whereas the miniaturized thermal shift assay provided an affinity of 1.1 μM. The known ligand pentosane polysulfate is reported to have a *K*_d_ of 11 μM with FGFR-1, as measured by ITC titration (Pantoliano et al. 1994), while the thermal shift assay, i.e., DSF, shows a similar binding ability of 5.5 μM. Thus, the reported thermal shift assay supports a reliable alternative for determining the interactions between proteins and small molecules.

1 $$ {K}_L^{Tm}=\frac{\mathit{\exp}\left\{-\Delta  {H}_u^{T0}/R\left[1/{T}_m-1/{T}_0\right]|+\Delta  {C}_{pu}^{T0}/R\left[ In\left({T}_m/{T}_0+{T}_0/{T}_m-1\right)\right]\right\}}{\left[{L}_{Tm}\right]}\kern0.5em $$

where

$$ {K}_L^{Tm} $$
ligand association constant at *T*_m_
*T*_m_: midpoint for the protein unfolding transition in the presence of ligand
*T*_0_: midpoint for the unfolding transition in the absence of ligand
$$ \Delta  {H}_u^{T0} $$
enthalpy of protein unfolding in the absence of ligand at *T*_0_
$$ \Delta  {C}_{pu}^{T0} $$
change in heat capacity on protein unfolding in the absence of ligand
[L_Tm_]: free ligand concentration at *T*_m_ ([*L*_Tm_]≅ [*L*]_total_ when [*L*]_total_ >>[Protein]_total_)
R: universal gas constant

DSF has a direct application in fragment-based ligand design (FBLD) due to the ease of use in high-throughput screening. In this approach, small molecule building blocks (100–150 Da) are potentially pooled (3–5 molecules per pool) and screened (Elkin et al. 2015; Valenti et al. 2019). Although these small molecular mass compounds are unlikely to possess high affinity by themselves, this pooled approach allows for a significant reduction in the number of experiments that need to be performed to screen a large library. Successful “hit” pools identified on the basis of a shift in *T*_m_ can then be examined in more detail to uniquely identify fragments of interest and hits can be grouped to provide a primary metric for lead compound optimization. This strategy also provides a high probability of adding blocks to the final scaffold of lead compounds (Mashalidis et al. 2013), and two recent examples of the use of DSF in lead discovery are provided below.

### DSF as a simple and robust mechanism to probe fragment-binding modes and suggests linking strategies

Tuberculosis (TB), caused by *Mycobacterium tuberculosis* (Mtb), remains one of the top 10 causes of death, and Mtb is the leading infectious agent (above HIV/AIDS) worldwide. In 2017, 10 million people developed TB resulting in 1.6 million deaths (World Health Organization 2018). Drug-resistant TB continues to be a public health crisis, and we still lack robust therapies to combat this burden. Consequently, new antitubercular agents that target TB with novel mechanisms are urgently needed. Biotin, also known as vitamin B_7_, is an essential cofactor for Mtb (Hayakawa and Oizumi, 1987). As Mtb produces biotin in order to support growth and proliferation, but this vitamin is present at very low concentration in human blood (Sassetti and Rubin 2003), therefore, targeting the biotin biosynthesis route intermediate by PLP-dependent transaminase (BioA) turns out to be a promising strategy (Mann and Ploux 2006). Dai and colleagues screened a Maybridge Ro3 fragment library with approximately 1000 compounds against BioA using DSF and discovered 21 “hit” compounds—identified as those that increased the *T*_m_ more than 2° (Dai et al. 2015). Subsequent X-ray diffraction data of cocrystals confirmed 6 fragment hits binding within the active site. The binding affinity and ligand efficiencies were cross-validated by ITC, giving a range between 7 and 42 μM in affinity and between 0.43 and 0.55 in ligand efficiencies, respectively. Comparison of all the available hits provided the basis for understanding the interaction mode of residues involved in the active pocket, leaving sufficient guidance for a lead sketch optimization consistent with the active site conformational states. Moreover, the scaffold of the small fragments found by DSF and crystallography also matched existing potent inhibitors previously reported (Park et al. 2015), further demonstrating that this strategy can be a reliable method for ligand screening.

**Fig. 2:**
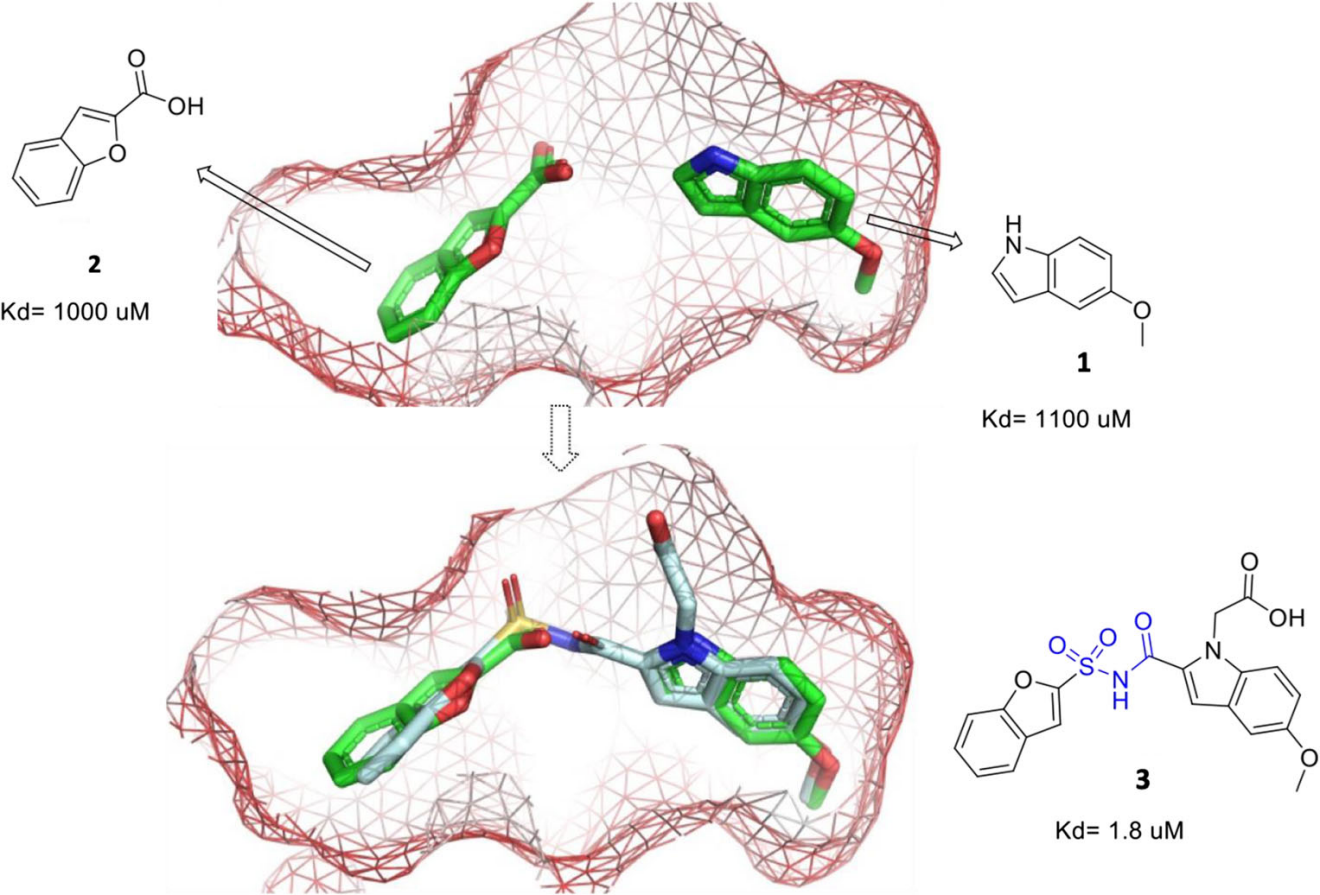
Fragments **1** and **2** soaked as a cocktail into the crystal of pantothenate synthetase. The two fragments are found to bind in distinct positions. Overlay of the linked lead compound **3** with fragments **1** and **2** in the active site of P1 of pantothenate synthetase. Fragments **1** and **2** shown as sticks in green. The benzofuran group is slightly rotated relative to fragment **2**, indicating that the stereochemical constraints of the linker do not allow this moiety to adopt its optimum conformation. Figures created by using PyMol, based on PDB entry 3IMG and 3IVX (Hung et al. 2009)

The same strategy was implemented by Hung’s team, targeting pantothenate synthetase (PS) of TB (Hung et al. 2009). Pantothenic acid (vitamin B_5_) plays an important role in fatty acid metabolism. It is formed through condensation of pantoate with β-alanine by pantothenate synthetase (PS), and blocking this pathway will likely impact the growth of Mtb (Sambandamurthy et al. 2002). In fragment screening via DSF, ligand **2** was identified from 1300 fragments with a Δ*T*_m_ of 1.6 °C (Fig. 2). This was further confirmed by WaterLOGSY NMR spectroscopy and ITC (*K*_d_ = 1 mM). The associated X-ray structure showed that **2** binds across the pantoate-binding pocket P1, extending further along the surface of PS, to a point 3.1 Å away from another binding site of ligand **1** in the same pocket. A test with both ligands soaked into crystals showed the presence of both fragments in the active site without clashes, in conformations similar to their individual binding modes (Fig. 2). Therefore, fragment linking and optimization were recruited to enhance binding properties, with different linkers based on the adjacent structures inside the pocket. Subsequently, lead compound **3**, which links fragments **1** and **2** by an acyl sulfonamide, showed a 500-fold stronger binding affinity than the individual fragments (Fig. 2).

### DSF combined with limited proteolysis in the identification of tankyrase inhibitors

**Fig. 3:**
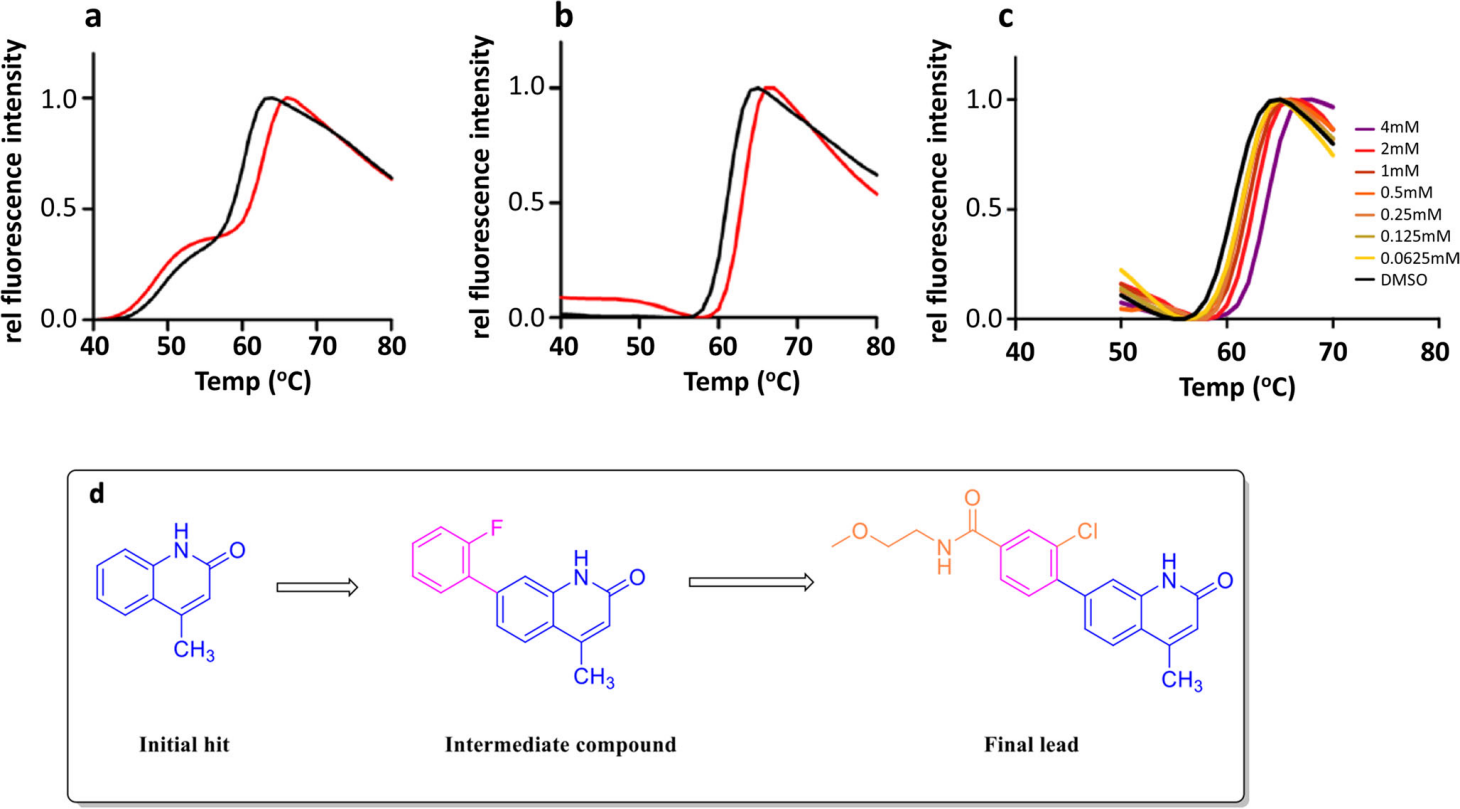
**a** Tankyrase 2 melting curves without chymotrypsination in the absence (black) and presence (red) of a stabilizing fragment. **b** Tankyrase 2 melting curves treated with chymotrypsin in the absence (black) and presence (red) of the same stabilizing fragment. **c** Concentration-dependent response for the stabilizing fragment with chymotrypsin-digested tankyrase. **d** The workflow of the final lead compound optimization from the initial hit to the end was guided by DSF. This figure was adapted with permission from Larsson et al. (2013). Copyright 2013 American Chemical Society

A fragment-based study performed by Larsson in 2013 gives a clear example of how DSF can be used to identify high-quality fragments followed by guiding the construction of a lead compound (Larsson et al. 2013). In this assay, the poly-ADP-ribosylating enzyme tankyrase was screened against a 500-compound fragment library (each present at 1 mM). To avoid oddly behaving compounds and minimize false-positive rates (i.e., pan-assay interfering compounds, PAINS) (Baell and Nissink 2018), identified hits are further validated to genuine “hits” by checking for a dose-dependent DSF response over a range of concentration (from 5 to 4000 μM). In the DSF screening of tankyrase 2, a “hit” melting profile was interpreted as those showing a two/multiple-state transition, which significantly complicated the fitting of *T*_m_ for weakly binding fragments (Fig. 3a). After adding chymotrypsin to perform an in situ digestion and remove less-ordered contaminants, they succeeded in simplifying the sigmoidal melting cure (Fig. 3b). Dose-response experiments then validated initial “hits” through an apparent increase in *T*_m_ upon elevated concentrations of an initial “hit” (Fig. 3c). Based on the cocrystal structure of TNKS2 with validated hits, various modifications of the hit fragment were proposed and evaluated. The 4-position methyl group was maintained as it protrudes down toward the catalytic glutamate, whereas changes in the 7-position group, which points toward the extended pocket responsible for adenosine binding, showed distinct differences when ligated to different functional groups. Starting with an initial fragment of 12-μM affinity, multiple rounds of modification and validation by DSF, SPR, enzymatic activity (IC_50_), and X-ray crystallography yielded a lead compound with an inhibition activity (IC_50_) of 9 nM and binding affinity (*K*_d_) of 16 nM against TNKS2. The elegant approach of limited proteolysis of the less stable (i.e., unbound) form of the target directly addresses one limitation of DSF—incomplete binding leading to multiple transitions in the thermal profile—amplifying weak binding. However, it is likely that such an approach will be highly dependent on the target under examination and may not be generally applicable.

In summary, the examples above both show that fragment-based drug discovery (FBDD) has become a mainstream choice for high-throughput screening for lead discovery of therapeutic interest (Congreve et al. 2008; Murray and Rees 2009) and that DSF has been validated as a robust option in preliminary screening in FBDD for more than 2 decades (Pantoliano et al. 1997). The use of DSF in fragment screening is facilitated by its low sample consumption—both in proteins and in chemicals—as well as the rapid determination of experimental Δ*T*_m_ determination—reducing labor-intensive work and providing simplified screening protocols.

### The use of DSF in buffer screening and optimization of protein stability and crystallization

In proteomics studies, inter-related biochemical, cellular, and physiological information is essential to reveal protein mechanisms. A major source of information is the use of structural, functional, and chemical genomics to characterize target proteins (Christendat et al. 2000). However, the common first step for all these approaches is the purification of the target protein, which remains challenging in many cases. On average, only 50–70% soluble protein and 30% membrane proteins from prokaryotes can be expressed in a recombinant form, and among those successfully expressed, only 30–50% can be purified in a homogeneous state (Christendat et al. 2000; Norin and Sundström 2002; Dobrovetsky et al. 2005). Eukaryotic proteins—including many biomedically interesting targets from humans—seem even more challenging (Banci et al. 2006).

Traditional solutions for protein production and purification mainly rely on the screening of recombinant hosts, encoding construct sequences, expression conditions, and then purification conditions (Gräslund et al. 2008; Rosano and Ceccarelli 2014; Wingfield 2015). In the last two steps, the addition of specific additives or changing buffer composition can significantly increase the solubility of recombinant proteins, as well as improving the thermal stability of the target to prevent protein unfolding or aggregation—even at a low temperature. There have been many reports (Sarciaux et al. 1999; Vedadi et al. 2006; Reinhard et al. 2013) showing that optimization of the purification conditions results in enhanced protein stability or solubility and it is not unreasonable to propose that buffer optimization should be seen as an integral part of any research project that relies on isolated protein samples. Even minor gains in protein stability can be significant in the context of process engineering, for example in the mass production of antibodies for therapeutic purposes.

One remarkable case is that of the recombinant protein dnaB, produced in *E*. *coli*. Initially, it was shown to be highly unstable in the purification buffer—even when stored at 0 °C, 90% enzymatic activity was lost within 30 min. In a stepwise screening process where specific chemical reagents (Mg^2+^, ADP, (NH_4_)_2_SO_4_, and glycerol) were added, 90% activity was retained after extensive storage at 60 °C in the optimal buffer. Furthermore, the new buffer helped the isolation of soluble dnaB at increased yields and subsequent crystallization (Arai et al. 1981). While this is undoubtedly an extreme example, this clearly shows the value of buffer optimization.

In the early years of structural genomics, a generally applied strategy was to use a default purification buffer for the majority of protein targets, with detailed optimization of sample buffer performed only to address pathological issues (aggregation, loss of activity, change in oligomeric state, etc.) (Mezzasalma et al. 2007). As shown below, this likely impacted the ultimate success of structural genomics projects, in which the growth of high-quality crystals from purified samples represented the major bottleneck. To address the issue of buffer optimization, Ericsson and coworkers developed a DSF-based screening system (comprised of different pH buffers, additives, heavy atoms, etc.) to test 25 different proteins expressed in *Escherichia coli* (Ericsson et al. 2006). The buffers consisted of a set of 23 different buffering agents at a concentration of 100 mM with a pH range from 4.5 to 9.0. Because each pH step is only 0.2 to 0.5 pH unit, it makes the screen wide enough for the majority of proteins investigated currently.

**Fig. 4:**
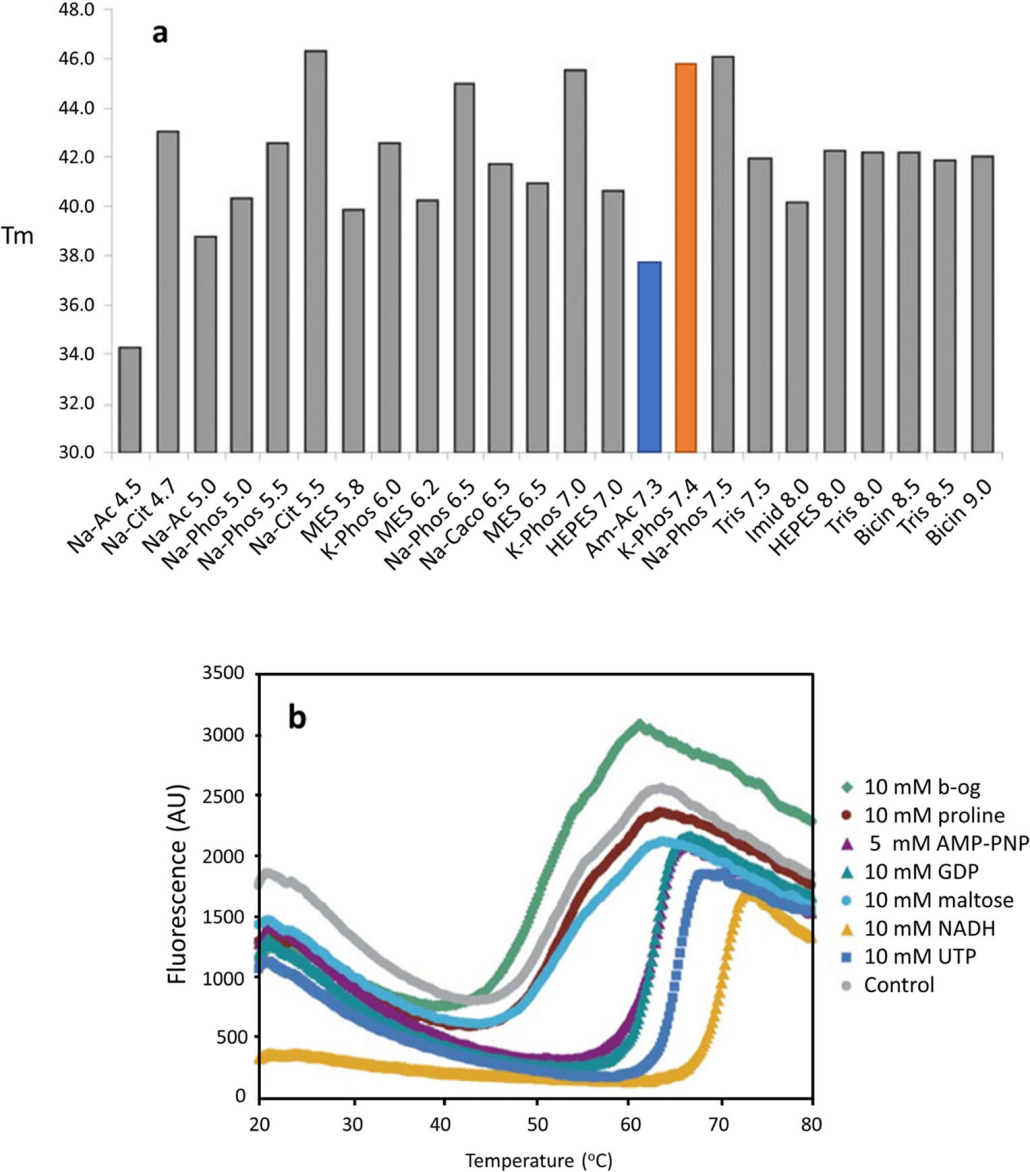
**a** Unfolding temperature of AC07 in various pH buffers of different compositions. Na-phosphate (red bar) and K-phosphate (blue bar) at a pH close to 7.4 showed a significant difference in *T*_m_. **b** Melting temperature curves of the protein AD21 screened against different additives. As an essential chemical needed in the proline biosynthetic pathway, NAD(P)H (yellow) showed a visible increase in thermal stability when incubated with the target protein. The figures are adapted from Ericsson et al. (2006). Copyright 2006 with permission from Elsevier

In some cases, protein *T*_m_ was dramatically influenced by a single pH buffer, correlated with a preference for specific ionic effects. For example, at pH 7, the *T*_m_ of protein AC07 in K-phosphate is 37 °C, whereas it is 46 °C in the presence of Na-phosphate (Fig. 4a). In order to decouple the influence of the choice of buffer and the final pH, a three-component buffer system (Newman 2004) was implemented, which allowed a wide range pH without altering the composition of buffer chemicals. The citric acid-Hepes-Ches (CHC) buffer, which covers the pH range from 4 to 10, can quickly identify the most favorable pH of target proteins. This work showed that the *T*_m_ of the targets examined followed a typical bell-shaped curve. For example, AD28 demonstrated lower temperature stability values at both low and high pH (pH = 4 and 10), with a maximum stability close to pH 6.4.

Combinations of the above buffer optimization with additives, such as heavy metals, or substrates/cofactors like NADH at optimal pH can further enhance protein thermal stability. For example, the addition of NADH was found to increase the melting temperature of AD21 significantly (Δ*T*_m_ ≈ 20 °C; Fig. 4b), which correlated with the previously known fact that it is an essential cofactor of AD21 in the catalysis of the last step in proline biosynthesis.

In summary, DSF screening of additives provided data to optimize the buffer conditions for crystallization screening (Reinhard et al. 2013). Additives that gave a positive thermal shift (*T*_m_) compared with control samples increased the protein crystallizing rate by 70%, while additives that showed destabilizing effects reduced the chance of getting crystals by around 50% compared with the control buffer. This observation strongly suggests a correlation between protein stability/solubility and crystallogenesis. For excellent in-depth reviews into the use of DSF to optimize crystallization buffers, the reader is referred to Boivin et al. (2013) and Reinhard et al. (2013).

**Fig. 5:**
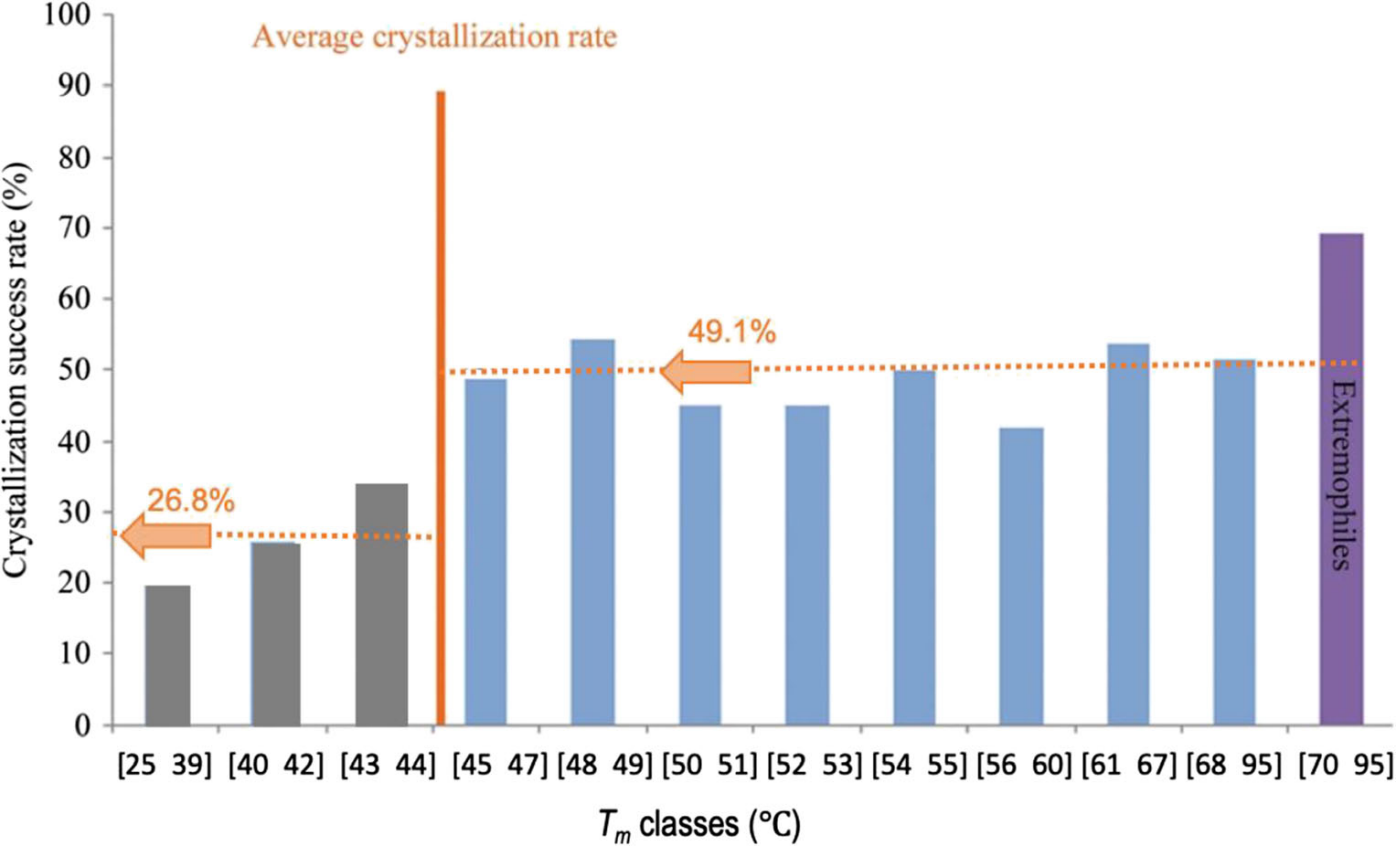
*T*_m_ and success rate in crystallization: all the samples were incubated for crystallization at 20 °C; the numbers above the bars indicate the success rate in crystallization of each class. The samples from extremophilic organisms consist of 12 proteins with *T*_m_ between 70 and 95 °C. The figure is adapted from Dupeux et al. (2011). Reproduced with permission of the International Union of Crystallography

Structural biology plays an important role in early-stage drug discovery, as the elucidation of the binding modes of “hit” compounds can provide essential information to drive downstream, lead compound development (de Kloe et al. 2009; Wang et al. 2019). While crystallization of proteins relies on a number of sample properties, with sample purity and homogeneity generally agreed to be the key determining factors (Giegi et al. 1994; Dale et al. 2003; Ericsson et al. 2006), thermal stability has also been shown to be a critical parameter in a successful outcome during crystallogenesis. In a study carried out by Dupeux et al. (2011), 657 different proteins were screened by DSF, then subjected to automated vapor-diffusion crystallization. Based on an analysis of the protein melting point (*T*_m_) and visually determined crystallization hits, the authors were able to draw clear inferences on the importance of thermal stability on the crystallization process. In this study, 437 of the 657 samples unfolded show clear and sharp temperature transitions. This behavior may be interpreted as the result of a sample population consisting of a single overall conformation, with relatively little conformational fluctuation around the “mean” fold—a scenario which is likely to be more conducive to crystallization than a sample with a high degree of conformational variation due to thermal mobility of its component elements. The average *T*_m_ for the ensemble of samples was 51.5 °C over a range of 25 to 95 °C (Fig. 5). Notably, proteins with a *T*_m_ of 45 °C or higher displayed a greater tendency to crystallize when incubated at 20 °C, with successful crystallization outcomes of 49.1%. For proteins with a *T*_m_ below 45 °C, the likelihood of crystal growth chance at 20 °C dropped to 26.8%. Additionally, a number of proteins with a *T*_m_ between 25 and 45 °C produced crystals at the lower temperature of 5 °C, where crystallization was initially unsuccessful at 20 °C. The study confirmed a previous observation that thermophilic proteins have higher rates of crystallization than those from mesophilic organisms, despite similar *T*_m_ values. In addition, a report from Szilágyi also implied that thermophilic proteins have a lower proportion of unstructured regions (Szilágyi and Závodszky 2000), with the inference that the disordered regions will hamper crystallization.

**Fig. 6:**
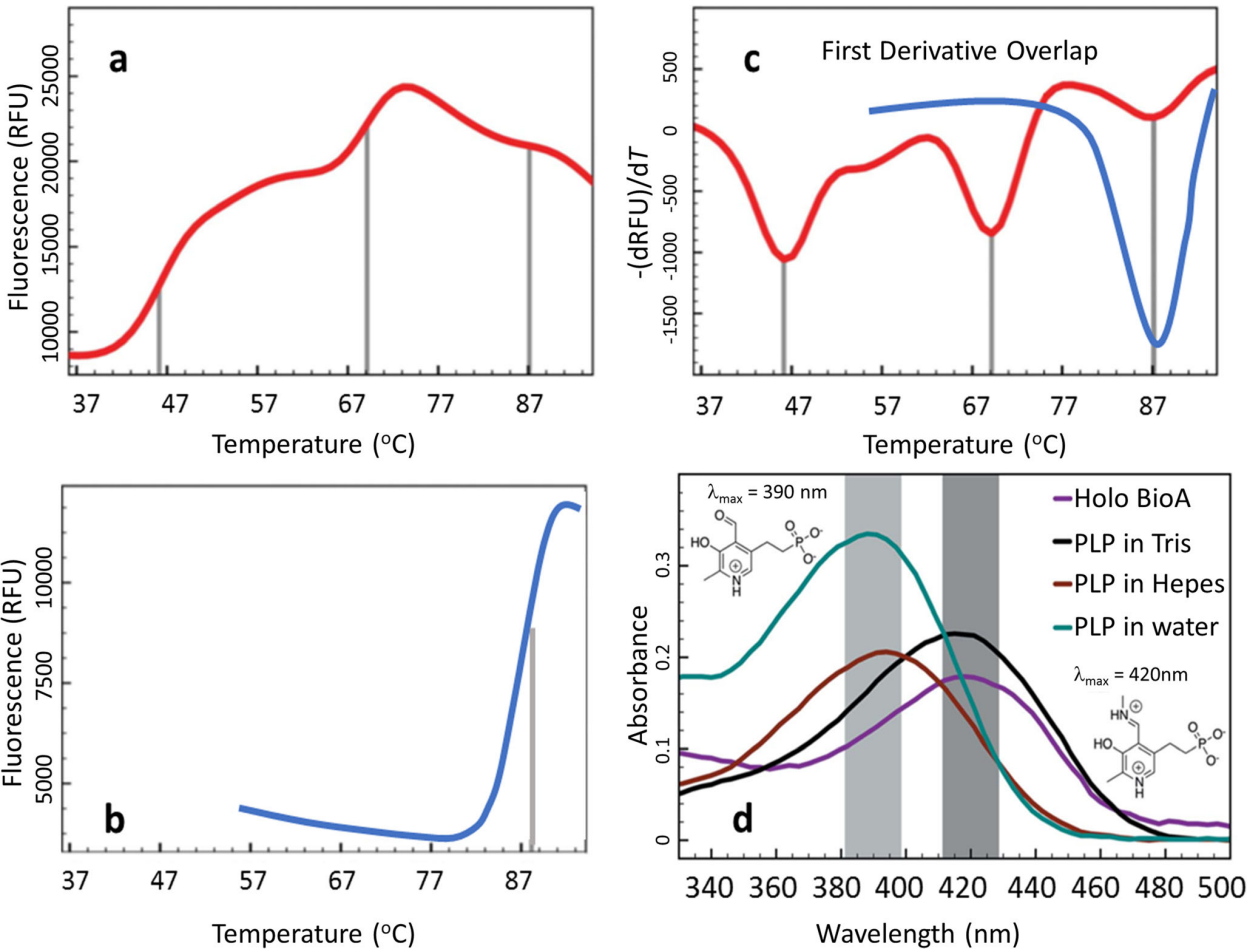
**a** DSF melting curves of BioA with PLP and Tris in both lysis and storage buffer, which shows multiple peaks during denaturing. **b** A sharp DSF melting curve of BioA with subsaturation of PLP; misfolded and apo peaks were eliminated after BioA was saturated with PLP, resulting in enhanced stability of BioA, with a *T*_m_ at 88 °C. **c** First derivative overlap of the corresponding melting curves. The red line indicates BioA in Tris buffer, with multiple transitions at 45, 68, and 86 °C, representing the misfolded, apo, PLP-bound BioA, respectively. The blue line represents BioA saturated with PLP for which the *T*_m_ was enhanced dramatically to 88 °C. **d** UV-Vis spectroscopy of PLP or PLP-BioA(holo) at various conditions; 400 μM PLP in water (cyan) has the same absorbance as in Hepes buffer (brown); PLP-bound BioA(holo) (purple) showed the same absorbance close to 420 nm as PLP in Tris buffer (black). The figures are adapted from Geders et al. (2012). Reproduced with permission of the International Union of Crystallography

As the thermal stability of a sample may influence its chances of crystallizing, it becomes clear that optimizing the sample buffer in which the protein is finally purified and concentrated prior to crystallization can provide benefit to structural biologists, and structure-based drug design in particular. In a typical DSF buffer screening experiment, the conditions (buffering agent, pH, additives, etc.) that result in the largest thermal shifts are often combined and the resulting buffer is then used for purification and crystallization. However, this process can be complicated when multiphasic unfolding behavior is encountered as it makes accurate *T*_m_ determination more difficult. A multiphasic unfolding curve typically indicates either the presence of multiple, independently folding, domains (Ionescu et al. 2008) or a heterogeneous state of the protein sample in solution (Choudhary et al. 2017), or ligand binding is not fully saturated with protein targets (Shrake and Ross 1992; Matulis et al. 2005), which may disrupt crystallogenesis and hinder protein functional characterization. Here, DSF can also be applied to guide sample preparation buffer screening for crystallization by replacing the buffer ingredients or ligands stepwise. Geders et al. reported a multiphasic unfolding behavior when his team attempted to crystallize pyridoxal 5-phosphate (PLP)–dependent transaminase BioA from *Mycobacterium tuberculosis* (Geders et al. 2012). During buffer optimization for crystallization, BioA displayed a multiphasic unfolding behavior without PLP; subsaturation of cofactors in the protein-cofactor system also yields a biphasic melting curve. The protein heterogeneity resulting from insufficient levels of cofactor PLP could potentially impact crystallization. To avoid the competition for PLP binding by other factors and to induce PLP saturation of BioA, DSF was used to study PLP binding. The initial buffers used in both lysis and purification (Dey et al. 2010) were Tris-based—generating a tri-phasic melting temperature curve with transitions at 45, 68, and 86 °C (corresponding to misfolded, apo, and PLP-bound BioA, respectively (Fig. 6a)). The sample also displayed significant precipitation at higher concentration levels. The electron density from a crystal grown from a Tris buffer showed no interpretable density for a bound PLP molecule. Replacing the Tris buffer with Hepes within the purification (both lysis buffer and final purification buffer) resulted in a decreased tendency for multiphasic melting curves, especially while Hepes completely replaced Tris in both lysis and purification buffer (Fig. 6b). This result suggested that the Tris buffer partially degraded the PLP, resulting in unsaturated PLP binding to BioA partially. This partial degradation was further supported by a UV-Vis spectroscopy assay, in which PLP in Tris buffer showed an absorbance maximum near 420 nm, similar to that shown by PLP in the Schiff base form instead of a free aldehyde (Fig. 6d). PLP in Hepes buffer showed absorbance at 390 nm, similar to that of PLP in water. By replacing Tris with Hepes in all purification buffers and adding increased concentrations of PLP, the multiphasic melting curves were replaced with a single, sharp transition curve with a *T*_m_ at 88 °C. These optimizations also improved the size and quality of the crystals obtained and also resulted in clear electron density for a bound PLP molecule. Thus, the DSF analysis correlated with heterogeneity and suboptimal crystallization outcomes. This example also highlights two complications in small molecule screening: firstly, the use of Tris (or primary amines which can form Schiff base with aldehydes) should be avoided with PLP-dependent proteins—and researchers should be aware of the potential for similar effects in other protein cofactors. Secondly, care should be taken when analyzing multiphasic DSF profiles, as they may be due to molecular interactions of the screen with the buffer, rather than the protein target.

**Fig. 7:**
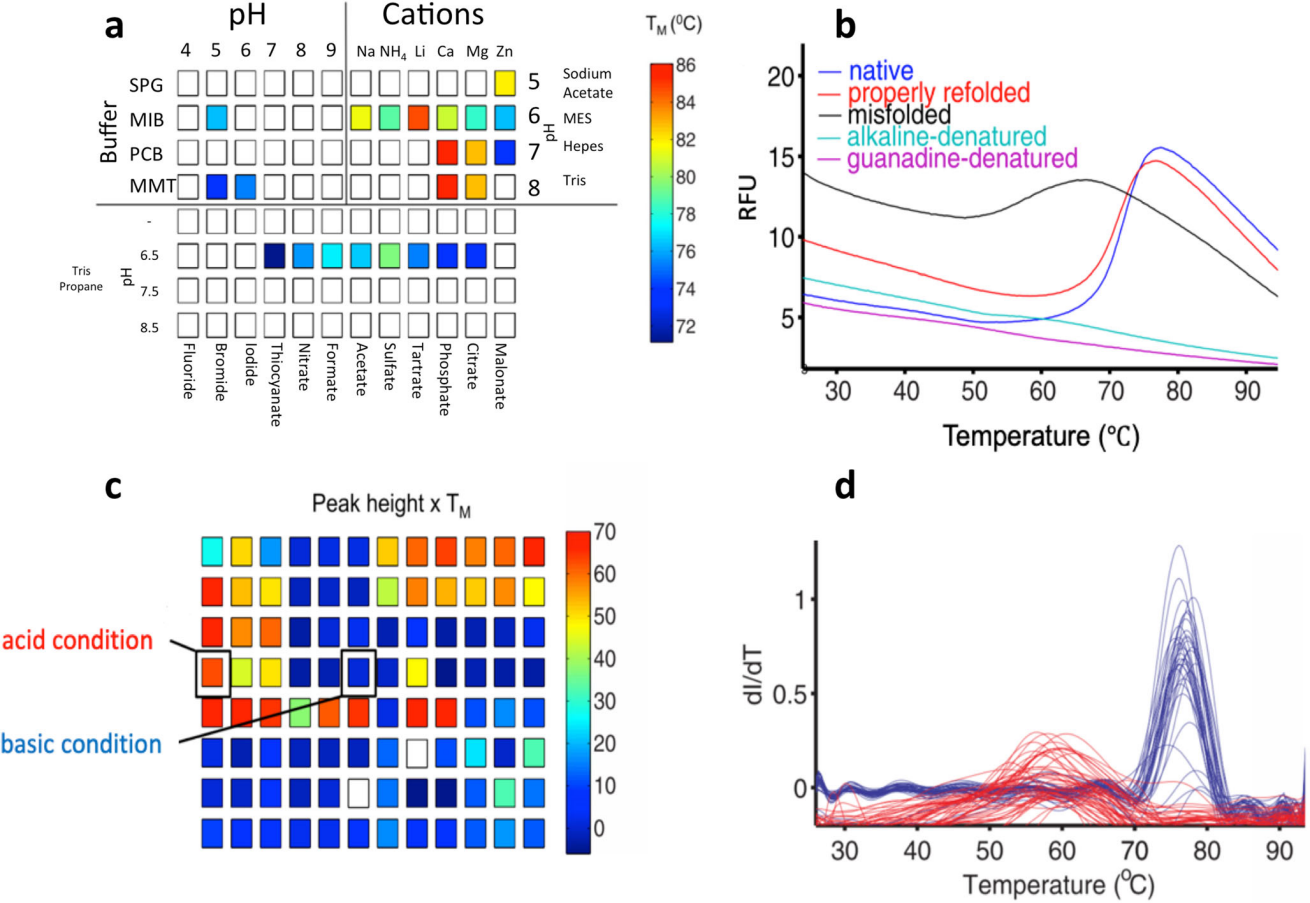
**a** The modified PACT screen in use in a refolding assay; three main parts consist of pH screen, cations, and anions in different combinations; the color indicates the *T*_m_ found in certain conditions. **b** Thermal melting profiles of pepsin in native, denatured, refolded, and misfolded states. **c** Peak height *T*_m_ in the PACK screen profile; the color indicates that under acid conditions, pepsin has a higher *T*_m_. **d** First derivatives of pepsin from the guanidine-solubilized dilution; populations in red correspond to the misfolded state, and blue is natively a folded state. The figures are adapted from Biter et al. (2016)

In biochemical or biomedical research, a well-folded protein structure with the correct activity is one of the critical factors for in vitro experiments. While numerous recombinant technologies exist to express proteins, greatly facilitating the understanding of proteomics in both prokaryotic and eukaryotic cells, the lack of suitable chaperones in *E*. *coli* (the most commonly used recombinant source) results in ~ 80% of these proteins misfolding into insoluble inclusion body without a defined fold or biological activity (Carrió and Villaverde 2002; Sørensen and Mortensen 2005; Gräslund et al. 2008; Rosano and Ceccarelli 2014). Moreover, refolding of proteins from inclusion bodies is an empirical art, with functionally related proteins of different construct designs or from different sources requiring significantly different conditions to support refolding. Thus, systematic and high-throughput compatible assays are needed to address this. In 2016, Biter and colleagues established a DSF-guided refolding method (DGR) to rapidly screen for the refolding of inclusion bodies, including proteins that contain disulfide bonds and novel structures with no preexisting model (Biter et al. 2016). The refolding trials used a PACT (pH, anion, cation testing) sparse matrix crystallization, leveraging the sparse matrix search of buffers to examine the large chemical space of biologically compatible buffers. Inclusion bodies were purified by centrifugation prior to solubilization in chaotropes (urea or guanidine) and the addition of a fluorescent dye (SYPRO Orange). Precipitants were excluded from the screen (Fig. 7a). The solubilized targets were incubated with components of the PACT screen for 2 h, centrifuged to remove any resultant precipitation/aggregation, and directly analyzed using DSF. Fluorescence data showing protein unfolding under DSF conditions was interpreted as corresponding to a condition that supported protein refolding. Due to the wide range in pH, cations, and anions, the PACT screen provided clear hints for pepsin refolding (Fig. 7c, d). For disulfide-containing proteins, such as lysozyme, the PACT screen conditions were supplemented with oxidized and reduced glutathione. The resulting thermal melting profile of the refolded lysozyme showed a clear *T*_m_ at 65 at pH 9 in the presence of equimolar GSH and GSSH.

Attempts to refold the novel proteins from inclusion bodies also succeeded in generating an improved yield of fibroblast growth factors 19 and 21, leading to crystals. When DGR was applied to the hormone Irisin, the success in refolding helped to generate an eight-dimer crystal form (Schumacher et al. 2013).

**Fig. 8:**
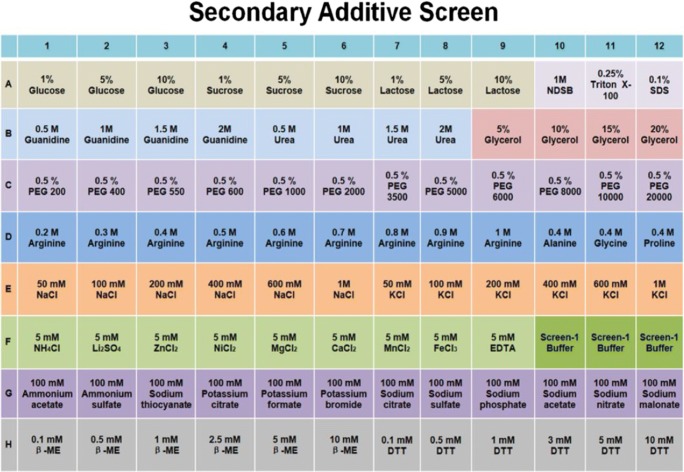
The composition of the secondary additive screen covers a wide range of sugars, detergents, salts, buffers, and reducing agents. This figure is adapted from Wang et al. (2017)

**Fig. 9:**
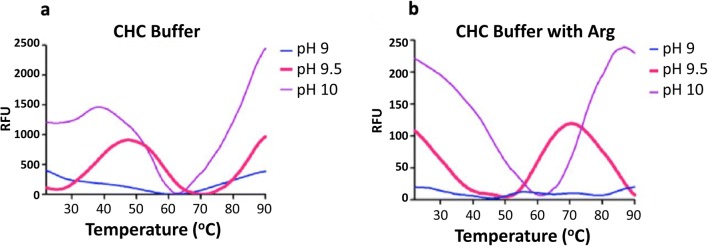
Melting transition of IL-17A in CHC buffer system at pH 9–10 in the absence (**a**) and presence (**b**) of arginine; both showed a typical sigmoidal melting curve at pH 9.5. The figures are adapted from Wang et al. (2017)

One year later, colleagues in our group expanded the DGR approach by investigating the refolding agent arginine and other additives in systematic buffer screens (Wang et al. 2017). Arginine has been widely used to suppress protein aggregation in refolding, and it can slow or prevent protein association reactions via weak interactions with the targets (Baynes et al. 2005; Arakawa et al. 2007), distinct from chaotropes such as urea or guanidine. Therefore, we designed two sequential screening kits to provide a general screening strategy. The primary screen is a combination of various pH buffers in the presence or absence of arginine at a concentration of 0.4 M. This can rapidly identify a suitable refolding pH while also screening for the effect of arginine in refolding. A secondary screen is then explored, by adding different sugars, detergents, osmolytes, PEGs, amino acids, concentration gradients of salt, and reducing agents, expanding on the PACT screen which mainly focuses on pH, anions, and cations (Fig. 8). This approach identified optimal refolding buffers for four different therapeutic target proteins from inclusion bodies expressed in *E*. *coli*, as well as identifying a final gel filtration buffer for storage or crystallization. A number of factors that affect protein refolding were revealed during this study, including the chemical composition of the buffer, refolding time, redox state, and the use of arginine as an inhibitor of aggregation. For example, DGR analysis of the refolding of interleukin-17A (IL-17A) gave obvious melting transition signals at pH 9.5 in CHC and CHES buffer—but not the MMT or MIB buffer system at the same pH—indicating that the compositions of the buffer have a significant effect. In the presence of arginine, the *T*_m_ increased from 40 to 60 °C, suggesting a more stable final product of the refolding process (Fig. 9). Refolding time also plays an essential role in all the assays, as data showed for all the proteins tested that the maximum efficiency appeared at a defined refolding time. The receptor-binding domain of hemagglutinin (HA-RBD) showed a clear melting curve when refolding was limited to 1 h, whereas the melting transition signal disappeared after 6-h incubation in refolding buffer. IL-17A needed extensive refolding time, requiring 15 h for an optimal DGR signal. Additionally, this data demonstrated that buffers optimized from the refolding process are not necessarily ideal for subsequent storage or crystallization—potentially as they stabilize an intermediate in the refolding process, rather than the final folded form.

### DSF applications for in vivo ligand: target interaction validation

A common issue in monitoring drug binding and efficacy during therapy is that the interactions between target proteins and drugs cannot be measured directly in cells and tissues. Validation methods normally study downstream cellular responses after multiple doses. Furthermore, some drugs tested may have good binding activity when incubated with target proteins but fail in clinical trials, with later research showing them to not act on the predicted target within cells (Auld et al. 2009; Schmidt 2010; Guha 2011). In 2013, Molina et al. (2013) introduced a new way to monitor the drug interactions inside cells by performing thermal shift assays on cells, lysates, or tissues, which is also based on ligand-induced thermal stabilization of target proteins, but no protein purification steps are needed. The cellular thermal shift assay (CETSA) functions by heating cells, whereby the proteins inside also unfold and precipitate—similarly to the in vitro approaches described above. After extract and centrifugation, the remaining soluble proteins were separated from the precipitate and quantified by Western blotting. Plotting the amount of soluble protein based on the Western blot signal strength provides the CETSA melting curve. In the preliminary study, dihydrofolate reductase (DHFR) and thymidylate synthase (TS) were selected as targets for the antifolate cancer drugs methotrexate and raltitrexed. Samples were exposed to either of the two drugs either as intact cells or as lysates. The result showed a distinct thermal shift increase for DHFR- or TS-treated cells compared with controls. To investigate drug concentration effects, an isothermal dose-response (ITDR) method has also been developed to assess binding of compounds. In this approach, cell lysate is aliquoted and exposed to different serial concentrations of the drug, while keeping the temperature and heating time constant. Following Western blotting, the signal strength can indicate when a higher drug concentration is needed for saturation, which is potentially more useful than commonly used half-saturation points (i.e., IC_50_, *K*_d_) which are related to affinity. Further research validated that the CETSA method can be applied as a reliable biophysical technique for studies of ligand binding to proteins in cells and lysates. In a recent report, Maji’s group screened a library with more than 2000 small molecules in order to identify inhibitors of CRISPR-Cas9, which could then be used for the precise control of CRISPR-Cas9 in genome engineering. CETSA was used to confirm a hit compound that disrupted the SpCas9:DNA interaction and decreased the *T*_m_ of SpCas9 by ~ 2.5 °C in compound-treated cells (Maji et al. 2019). In another structure-based design of a small molecule to target the interaction of menin-MLL in leukemia, an irreversible, highly potent chemical M-525 was also confirmed by CETSA in a cellular assay (Xu et al. 2018). The covalent-binding compound enhanced the thermal stability of menin in both MV4;11 and MOLM-13 cells; the concentration of M-525 used here was as low as 0.4–1.2 nM. Furthermore, CETSA also showed that the compound specifically targeted menin, and no effect was detected on another MLL-binding protein WDR5.

## Conclusion

DSF constitutes a robust biophysical technique for studying protein stability in a particular environment, either within selected buffer conditions or when (partially) saturated with ligands of interest. The protein unfolding thermodynamic parameter Δ*T*_m_ is monitored as the primary indicator to justify stability changes of the target protein, no matter whether targets were in a purified form, in lysate, cells, or even tissues. Newly emerged label-free nanoDSF approaches especially obviate the need for dyes, allowing the same approach to be applied to membrane protein research, simultaneously addressing problems caused by the interaction between dye and the hydrophobic surface of proteins, or the detergent additives applied and interactions between the dye and other molecules in a screen. Over the almost two decades since it first appeared, the DSF technique has been used to characterize the thermal properties of numerous proteins, aided by low sample consumption and high throughput—making DSF suitable for optimizing buffer ingredients in crystallization, as well as screening large ligand libraries. In terms of ligand-binding validation, although many successful cases have been reported in the literature, it is still important to be aware that this correlation typically occurs for similarly structured compounds within a series, and stubbornly pursuing fragment hits on the basis of significant thermal shifts may mislead further optimization. It should also be borne in mind that ligands can interplay with both the folded and unfolded states of target proteins, and a negative shift in melting temperature does not exclude binding to the native state. Unlike titration-based techniques such as ITC, MST, and SPR in which interaction behaviors of receptors rely on different serial concentrations of ligands and end-point measurements, DSF is sensitive to all stages along a binding pathway, complicating its use to determine the affinity of molecules toward mobile protein receptors. Nevertheless, the robustness and applicability of DSF to address various problems across such a wide range of sample types should ensure its status as a central technology of modern drug discovery.
